# Invasive mucinous adenocarcinoma of the lung: Serial CT findings, clinical features, and treatment and survival outcomes

**DOI:** 10.1111/1759-7714.13674

**Published:** 2020-10-05

**Authors:** Kyongmin Sarah Beck, Yeoun Eun Sung, Kyo Young Lee, Dae Hee Han

**Affiliations:** ^1^ Department of Radiology, Seoul St. Mary’s Hospital, College of Medicine The Catholic University of Korea Seoul The Republic of Korea; ^2^ Department of Hospital Pathology, Seoul St. Mary's Hospital, College of Medicine The Catholic University of Korea Seoul The Republic of Korea

**Keywords:** CT, invasive mucinous adenocarcinoma, lung adenocarcinoma, non‐small cell lung cancer

## Abstract

**Background:**

Invasive mucinous adenocarcinoma (IMA) of the lung is a rare and distinct subtype of adenocarcinoma that can appear as airspace opacities on computed tomography (CT). In daily practice, we have occasionally encountered spontaneous regression of airspace opacities (SRAs) without treatment on serial CTs in patients with IMAs, which has not previously been described in the literature. Here, we describe serial CT findings with emphasis on SRAs in relation to clinicopathological features and treatment outcomes in patients with IMAs.

**Methods:**

A total of 46 patients with pathologically‐confirmed IMAs of the lung from January 2013 to June 2018 were included. Serial CT scans were reviewed and the patients were classified into SRA and no‐SRA groups according to the presence of SRA. Radiological features, clinicopathological characteristics, and treatment outcomes were compared between the SRA and no‐SRA groups.

**Results:**

A total of 32 patients were included in the no‐SRA group and 14 patients in the SRA group. IMAs in the SRA group were mostly pneumonic (*P* < 0.001), larger (*P* < 0.001), multifocal (*P* = 0.001), and showed higher stage (*P* < 0.001) on initial CT. Of seven patients who died during follow‐up, six were from the SRA group (*P* < 0.001). Mean overall survival for all IMAs was 86.6 months (range, 0–110 months), and the SRA group showed significantly worse overall survival (*P* < 0.001).

**Conclusions:**

IMAs of the lung showing SRAs on serial CTs are larger and multifocal, and tend to be pneumonic in type on initial CT. Patients present at a higher stage of disease, with higher mortality rate and reduced overall survival.

**Key points:**

**Significant findings of the study:**

- Invasive mucinous adenocarcinomas (IMAs) of the lung can show spontaneous regression of airspace opacities (SRAs) on serial CTs, without being correlated to the administration of anticancer drugs. IMAs that showed SRAs demonstrated reduced overall survival in patients.

**What this study adds:**

- When airspace opacities show regression on CT, IMA should still be included in the differential diagnosis. A more careful application of RECIST 1.1 is needed in the assessment of tumor response of IMAs.

## Graphical abstract

Invasive mucinous adenocarcinomas (IMAs) of the lung can show spontaneous regression of airspace opacities (SRAs) on serial CTs, not correlated with the administration of anticancer drugs. IMAs that show SRAs demonstrated reduced overall survival in patients. When airspace opacity shows regression on CT, IMA should still be included in the differential diagnosis and a more careful application of RECIST 1.1 is needed in the assessment of tumor response of IMAs.

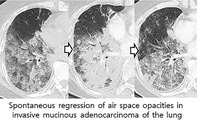

## Introduction

**Figure 1:**
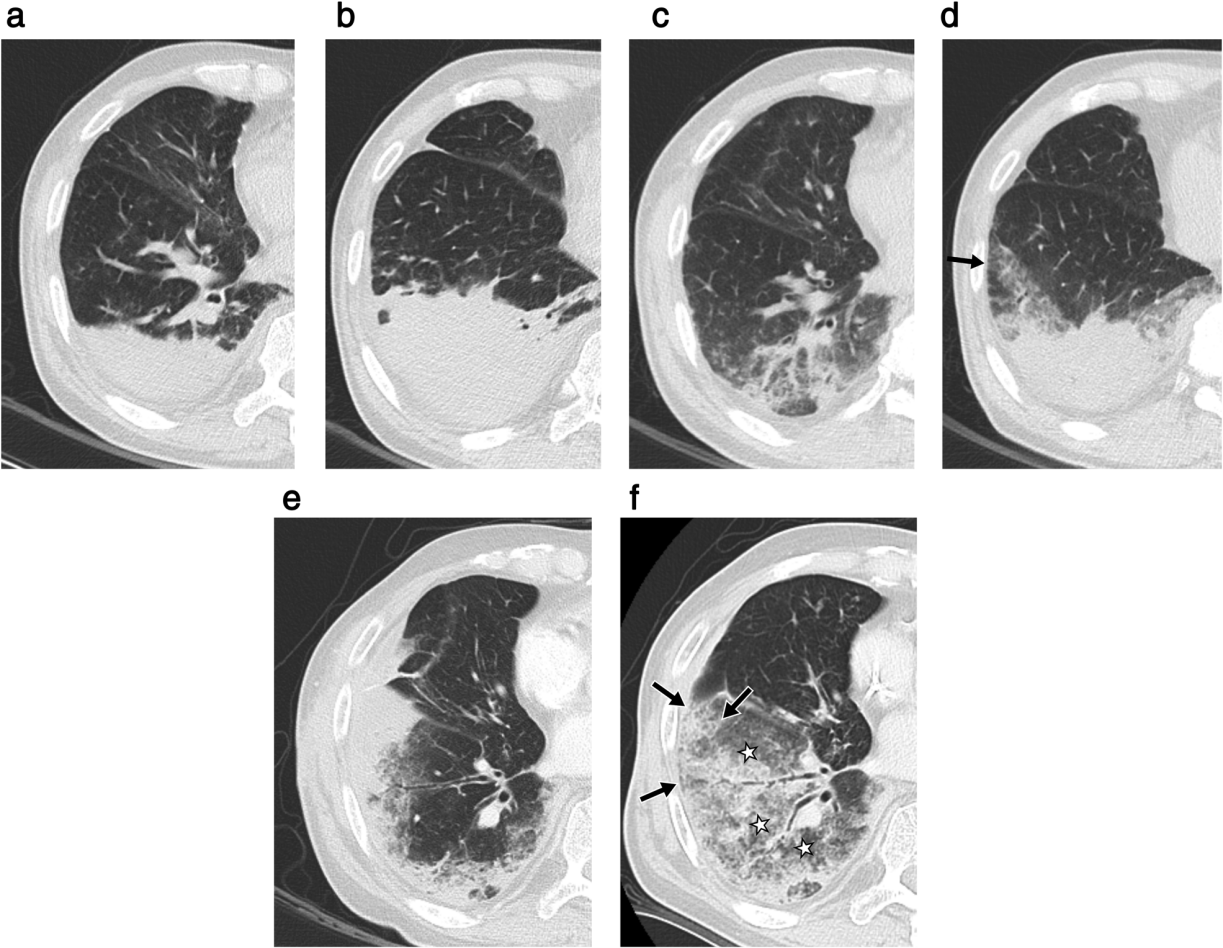
Axial chest CT images (section thickness, 3.0 mm) of a 77‐year‐old male with pneumonic‐type invasive mucinous adenocarcinoma showing two types of spontaneous regression of airspace opacities (SRA). (**a**, **b**) Initial chest CT showed consolidation in the dependent portion of right lower lobe. (**c**) Chest CT scan taken four months after the initial scan showed decreased extent and density of the consolidation. At a slightly caudal level (**d**) a new patchy ground‐glass opacity (arrow) is seen near the slightly regressed consolidation in the right lower lobe. The patient received antibiotics for three weeks after the initial scan but had not received any anticancer therapy. Although the lesion was later proven to be invasive mucinous adenocarcinoma, regression of airspace opacities without anticancer therapy was observed, and this was termed as SRA. (**e**) Patchy peripheral consolidation in the right lower lobe was seen on chest CT taken about a year after the initial scan. (**f**) The patient then received one cycle of cisplatin‐based combination chemotherapy with doxorubicin, and the post‐chemotherapy follow‐up chest CT scan revealed decrease in density and extent of the peripheral consolidation (black arrow), whilst also showing new areas of ground‐glass opacity (stars). This pattern of change after anticancer therapy was also considered to be SRA, because increase or decrease in extent of airspace opacities appears to occur regardless of the use of anticancer therapy.

Invasive mucinous adenocarcinoma (IMA) of the lung is a rare and distinct subtype of lung adenocarcinoma, previously known as bronchioloalveolar carcinoma (BAC).^1^ The distinct imaging findings of IMA, which appear as airspace opacities including both consolidation and ground‐glass opacities (GGO), and can mimick infectious pneumonia, are well known. However, IMA can also appear as a localized mass or a nodule, indistinguishable from other non‐small cell lung cancers (NSCLCs).^2^, ^3^ During our daily practice, we often encounter spontaneous regression of airspace opacities (SRA) in patients with IMA, or concomitant increase and decrease in the extent of different airspace opacities, which is seemingly not correlated with the administration of anticancer drugs (Fig 1). Although there have been many studies on IMAs with regard to their imaging findings, serial CT findings describing the changes in the extent of airspace opacities over time and in relation to the use of anticancer drugs have, to our knowledge, not been previously described in the literature. We found one case report describing “fluctuating extent of consolidation” in an IMA patient,^4^ similar to our experiences; however, there are no systematic scientific studies on this phenomenon.

There have been many studies on the prognosis and imaging findings of IMAs, most of which involve cases of IMA resection.^5^, ^6^, ^7^, ^8^, ^9^ These studies showed that patients with resected IMA had poorer prognosis compared to those with resected nonmucinous adenocarcinomas^5^, ^6^ or the resected solitary (nonpneumonic) type of IMA had a worse outcome than resected pneumonic type of IMA.^7^, ^8^, ^9^ Another study which compared the clinical course of stage IV IMAs to nonmucinous adenocarcinomas, also concluded that stage IV IMA patients showed much worse prognosis than those of their nonmucinous counterparts.^10^ However, the significance of SRA in relation to prognosis or clinical features of IMA has yet to be investigated, and should preferably include both IMA resected and unresected cases in order to gain a more comprehensive understanding.

Therefore, the purpose of this study was to describe the serial imaging findings with emphasis on SRAs, in relation to clinicopathological features and treatment outcomes of patients with IMA.

## Methods

The institutional review board at our hospital approved our retrospective study with a waiver of informed consent (IRB number: KC19RESI0250).

### Study population

We searched the electronic medical records for pathology reports of patients with “mucinous adenocarcinoma” or “IMA” of the lung from January 2013 to June 2018. A total of 47 cases were identified; however, one patient without any available follow‐up CT scan was excluded. In total, 46 patients with initial chest CT and more than one follow‐up CT scan were included.

The following clinical data were obtained from the electronic medical records: age, sex, smoking history (never smokers, former smokers, and current smokers), and packyear smoking history (defined as the number of cigarette packs smoked per day multiplied by the number of years of smoking). Diagnosis and treatment methods, overall survival (OS), and progression‐free survival (PFS) were also assessed.

### Radiological analysis

**Figure 2:**
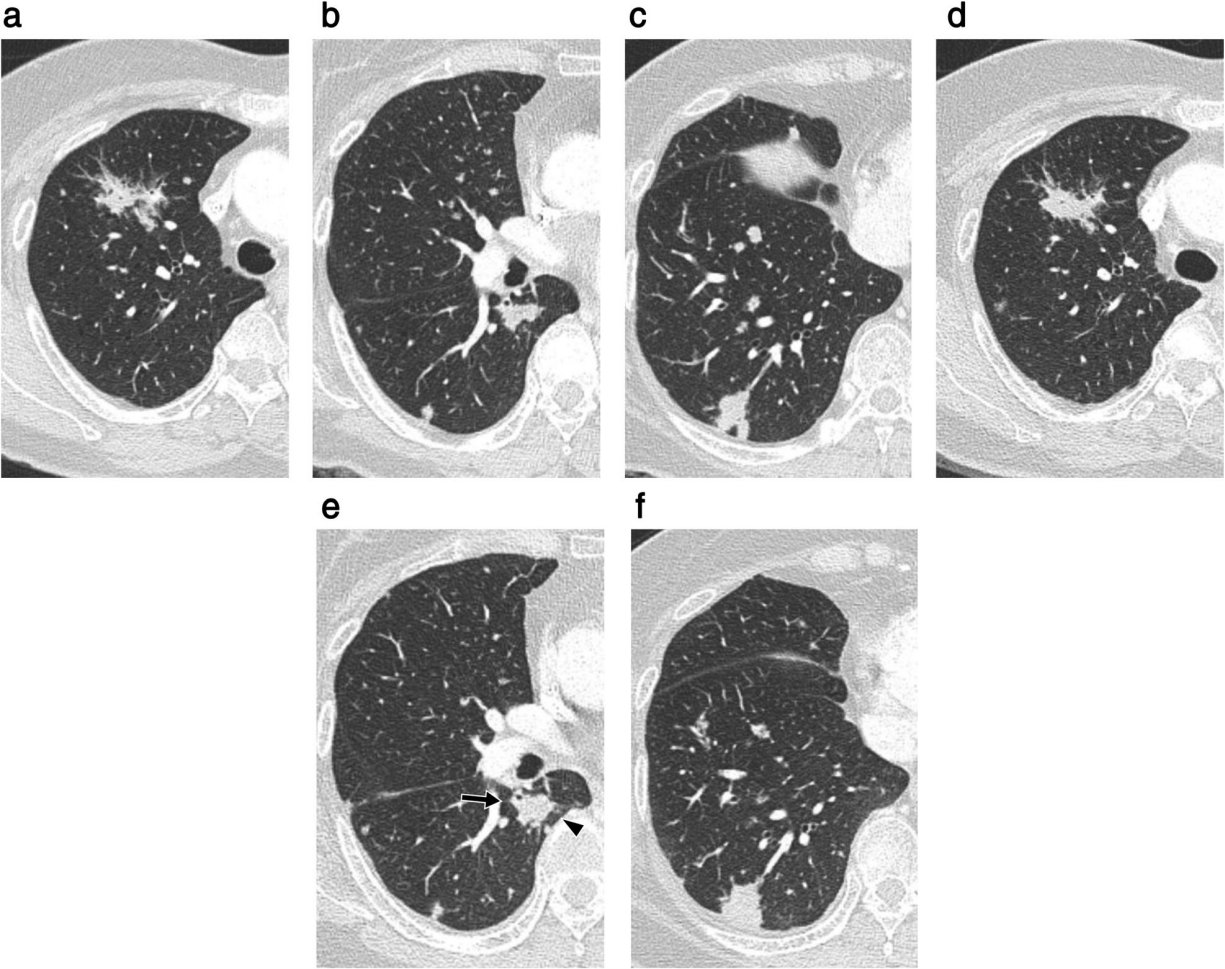
Axial chest CT images (section thickness, 3.0 mm) of a 64‐year‐old female patient with nonpneumonic‐type multifocal invasive mucinous adenocarcinomas in both lungs demonstrated spontaneous regression of airspace opacities (SRA). (**a**–**c**) Chest CT scan revealed (**a**) an irregular peribronchial lesion with surrounding ground‐glass opacity in the right upper lobe and (**b**, **c**) two irregularly‐shaped nodular cancer lesions in the right lower lobe. (**d**–**f**) After receiving two cycles of cisplatin‐based combination chemotherapy with doxorubicin, follow‐up chest CT showed (**d**) slightly decreased size of the peribronchial lesion in the right upper lobe with regression of the perilesional ground‐glass opacity, while showing (**e**) change in shape of another lesion in the right lower lobe – decreased in one portion (arrow) and increased in another portion (arrowhead). (**f**) Another irregularly‐shaped lesion in the right lower lobe had increased in size. This pattern of change after anticancer therapy was also considered as SRA, because increase or decrease in extent of airspace opacities appears to occur regardless of the use of anticancer therapy.

CT scans were reviewed by two chest radiologists (K.S.B. and D.H.H with five and 20 years of experience in chest imaging, respectively) and a conclusion reached for each case by consensus. First, initial CT scans of the patients, defined as the very first CT of the patient available on our PACS system with identifiable cancer lesion on CT, were reviewed. We classified the CT types of IMA (nonpneumonic vs. pneumonic), based on the dominant presenting pattern on initial CT, as in other studies.^5^, ^7^, ^8^ Nonpneumonic type was defined as a localized lesion with a definable shape, whereas pneumonic type was defined as amorphous air space opacities without definable shape or parenchymal distortion (Figure S1). Other CT features, such as tumor size (size of the largest lesion if multifocal), absence or presence of GGO component (in or around the lesion), distribution (solitary or multifocal), margin of the largest lesion, or pleural effusion were also assessed. Then, serial follow‐up CT scans of the patients were reviewed with emphasis on changes over time and in relation to medical or surgical treatment. For each patient, we checked the dates of follow‐up CTs and recorded whether the patient had received anticancer therapies (including cytotoxic chemotherapy, target therapy, and immunotherapy) between each follow‐up CT. We analyzed interval changes of the primary cancer lesion(s) and other airspace opacities with special interest on “SRA” by comparing it with the immediately previous CTs. We defined SRA as showing either: (i) decrease in extent and/or density of airspace opacities on post‐chemotherapeutic follow‐up CT in one or more area(s) with concomitant increase in extent and/or density of airspace opacities in other areas on a given CT; or (ii) regression of airspace opacities in one or more areas on follow‐up CT without anticancer therapy (Figs 1 and 2). We then classified patients into two groups: those who had shown SRA once or more during the whole follow‐up duration (SRA group) and those who had never shown SRA during the entire follow‐up duration (no‐SRA group).

### Pathological analysis

The surgically resected lung specimens were routinely fixed in 10% formalin solution, and embedded in paraffin, with 4 μm sectioning, and hematoxylin & eosin (H&E) staining. If surgical specimens were not available, needle biopsy specimens were evaluated. All H&E slides were reviewed by two thoracic pathologists (K.Y.L. and Y.E.S. with 37 and six years of experience in histopathological interpretation, respectively). IMA was diagnosed using the established criteria: tumor cells that characteristically show a goblet and/or columnar cell morphology with abundant intracytoplasmic mucin and small basally oriented nuclei.^11^

**Figure 3:**
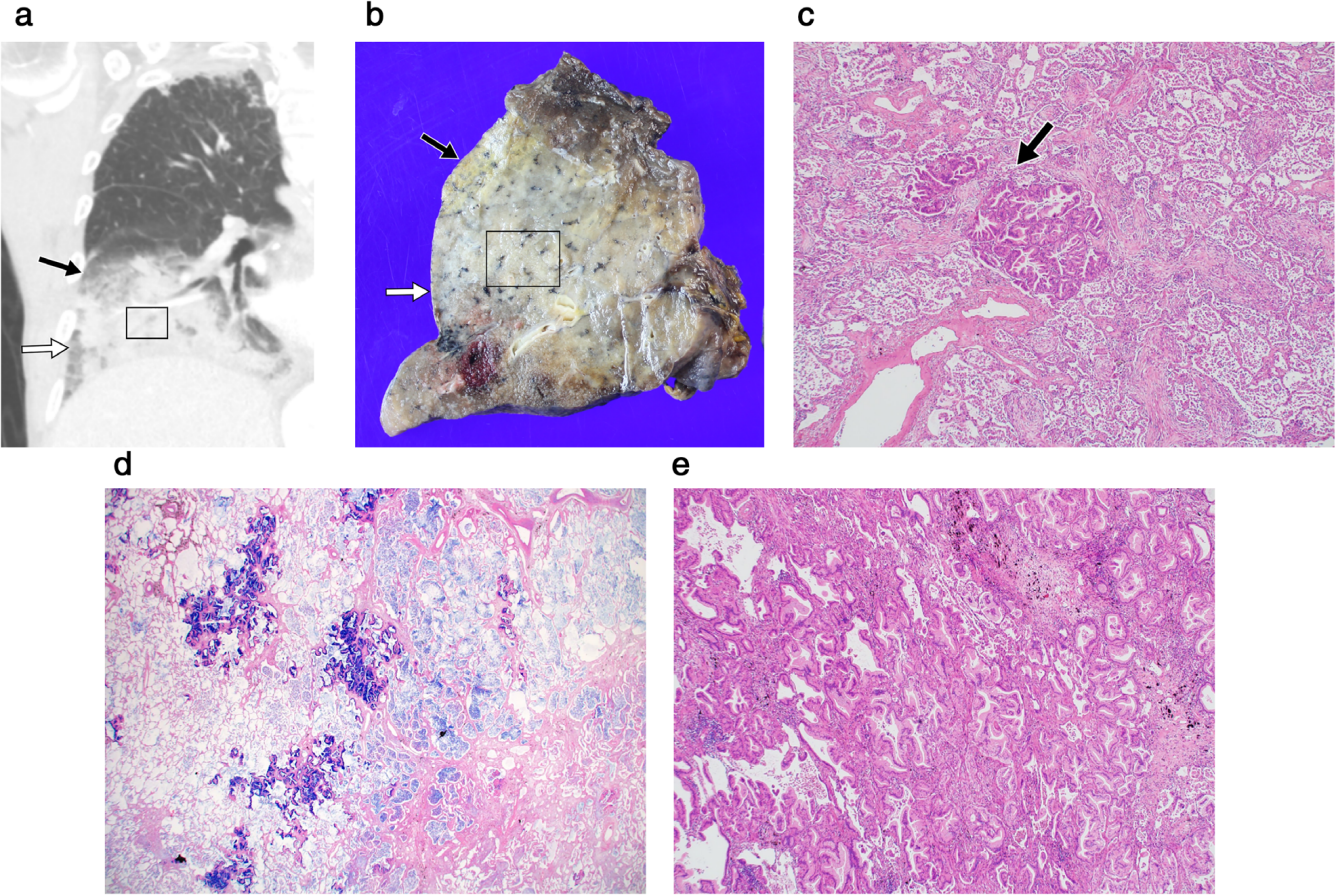
(**a**) Coronal reconstruction chest CT image (section thickness, 3.0 mm) of a 77‐year‐old male patient with pneumonic‐type invasive mucinous adenocarcinoma showed areas of ground‐glass opacity (black and white arrows) as well as dense consolidation (black rectangle) in the right lower lobe. (**b**–**e**) Lobectomy specimen of the same patient (**b**) showed matching areas of ground‐glass opacity (black and white arrows) and consolidation (black rectangle) seen on the CT image. Although similarly seen as ground‐glass opacity on CT, the areas indicated by a black arrow (**c**) and white arrow (**d**) differed on microscopic examination; the former (**c**) consists mostly of organizing pneumonia, intra‐alveolar infiltration of macrophages with mucinous material, and occasional invasive mucinous adenocarcinoma nests (black arrow) (hematoxylin and eosin stained, x40); the latter (**d**) mostly shows mucinous material, with multiple foci of invasive mucinous adenocarcinoma component (Alcian blue, pH 2.5, stained, x12.5). Photomicrograph of the area in the black rectangle (**e**), seen as consolidation on CT, shows areas of densely packed tumor glands with interstitial fibrous thickening (hematoxylin and eosin stained, x40).

Because SRA could only be confirmed on CT after the airspace opacities had already regressed, direct pathological analysis of SRA was not possible. Instead, we analyzed the available surgical specimens of IMAs in the SRA group to determine the pathology of SRA. This was done by matching the airspace opacities seen on CT and the gross specimen, considering the shape and location on CT as shown in Fig 3. Five patients from the SRA‐group underwent surgical resection and their surgical specimens were reviewed. The specimens all consisted of the pneumonic‐type IMAs, which presented as consolidation and GGO on CT. One radiologist (K.S.B.) and two pathologists (K.Y.L. and Y.E.S.) made consensus decisions on correlation.

Molecular studies, including epidermal growth factor receptor (*EGFR*) and *KRAS*, were performed in patients who gave their consent to the tests being performed. A total of 40 cases of *EGFR* and 16 cases of *KRAS* were ultimately included. Formalin‐fixed paraffin‐embedded (FFPE) specimens were used to extract DNA, using Maxwell 16 Tissue DNA Purification Kit (Promega, USA). A portion of *EGFR* detection (17 out of 40 cases) and *KRAS* detection was done by Sanger sequencing, using BigDye terminator v3.1 cycle sequencing kit (ThermoFisher Scientific). The remainder of *EGFR* detection was done with Applied Biosystem (ABI) 7500 Real time PCR and PNAClamp mutation detection kit (PANAGENE, Daejeon, Korea) according to the protocol. The PCR results were analyzed by PNAClamp Analyzer.

### Statistical analysis

Clinicopathological and radiological factors were compared between the SRA and no‐SRA groups by adopting the χ^2^ test, the Fisher's exact test, or the student's *t‐*test. Progression‐free survival (PFS) and overall survival (OS) were estimated by using the Kaplan‐Meier method, and the log‐rank test was used to evaluate the differences between the SRA and no‐SRA groups. Statistical analyses were performed with commercially available software (SPSS version 24.0; SPSS, Chicago, Ill). A *P*‐value less than 0.05 was considered statistically significant.

## Results

### Clinical characteristics and survival and treatment outcomes

Of the 46 patients included in the study (22 men and 24 women; mean age, 66.6 years; range, 26–82 years), 32 patients (69.6%) were classified as the no‐SRA group and 14 patients (30.4%) were classified as the SRA group. Their detailed clinical characteristics are summarized in Table 1. The SRA group presented with significantly higher overall stage (*P* < 0.001) and higher T (all patients in the SRA group were T4) and M classifications (*P* < 0.001 and *P* < 0.001, respectively). There were 12 stage I and 10 stage II patients all from the no‐SRA group, and the SRA group was comprised of only stage III and IV patients.

**Table 1:** Patient characteristics

	Total (*n* = 46)	No‐SRA (*n* = 32)	SRA (*n* = 14)	*P*‐value
Mean age ± SD, years	66.6 ± 11.1	65.0 ± 11.8	69.6 ± 9.5	0.224
Sex, n (%)				0.301
Male	22 (47.8)	13 (40.6)	8 (57.1)	
Female	24 (52.2)	19 (59.4)	6 (42.9)	
Smoking history, n (%)				0.434
Never	22 (47.8)	17 (53.1)	6 (42.9)	
Former	17 (37.0)	10 (31.3)	7 (50.0)	
Current	7 (15.2)	5 (15.6)	1 (7.1)	
Pack‐year, mean ± SD	15.4 ± 22.1	10.4 ± 16.7	23.8 ± 29.6	0.131
Stage				<0.001^*^
1	12 (26.1)	12 (37.5)	0 (0.0)	
2	11 (23.9)	10 (31.3)	0 (0.0)	
3	9 (19.6)	6 (18.7)	3 (21.4)	
4	14 (30.4)	4 (12.5)	11 (78.6)	
T classification, n (%)				<0.001*
T1	13 (28.3)	13 (40.6)	0 (0.0)	
T2	9 (19.6)	9 (28.2)	0 (0.0)	
T3	6 (13.0)	5 (15.6)	0 (0.0)	
T4	18 (39.1)	5 (15.6)	14 (100.0)	
N classification, n (%)				0.359
N0	39 (84.8)	27 (81.3)	12 (85.7)	
N1	1 (2.2)	0 (0.0)	1 (7.15)	
N2	4 (8.7)	3 (9.4)	1 (7.15)	
N3	2 (4.3)	2 (6.3)	0 (0.0)	
M classification, n (%)				<0.001*
M0	31 (67.4)	28 (87.5)	3 (21.4)	
M1	15 (32.6)	4 (12.5)	11 (78.6)	
Management, n (%)				<0.001*
Curative resection	30 (65.2)	27 (84.4)	3 (21.4)	
Stage I	12	12	0	
Stage II	10	10	0	
Stage III	8	5	3	
Palliative treatment				
Stage IV	13 (28.3)	4 (12.5)	9 (50.0)	
No treatment	3 (6.5)	1 (3.1)	2 (14.3)	
Death, n (%)	7 (15.2)	1 (3.1)	6 (42.9)	0.001*

*P < 0.05 is indicated by bold italics.

SD, standard deviation; SRA, spontaneous regression of airspace opacities; Y, year.

Of the 46 IMA patients, 30 patients underwent curative resection (22 lobectomies, one lobectomy and segmentectomy, four segmentectomies, and three wedge resections), 13 patients received palliative treatments (such as chemotherapy, target therapy, immunotherapy, lobectomy done with palliative intent, or radiotherapy), and three patients did not receive any treatment (two patients were transferred to other hospitals and one patient refused chemotherapy).

Of the 32 no‐SRA group patients, 27 (84.4%) patients (12 in stage I; 10 in stage II, and five in stage III) received curative resection and four stage IV patients received palliative treatment. Of the 12 SRA group patients, three (21.4%) stage III patients received curative resection and nine stage IV patients received palliative treatment (*P* < 0.001). After curative resection, three (11.5%) recurrences (each from stages I–III) in the no‐SRA group and three (100.0%) recurrences (all from stage III) in the SRA group were noted.

Two of the four patients who received palliative treatment in the no‐SRA group showed progression, and all nine patients who received palliative treatment in the SRA group eventually showed progression. The median PFS for the no‐SRA group was 24.0 months and that for the SRA group was 4.0 months (*P* = 0.117).

**Figure 4:**
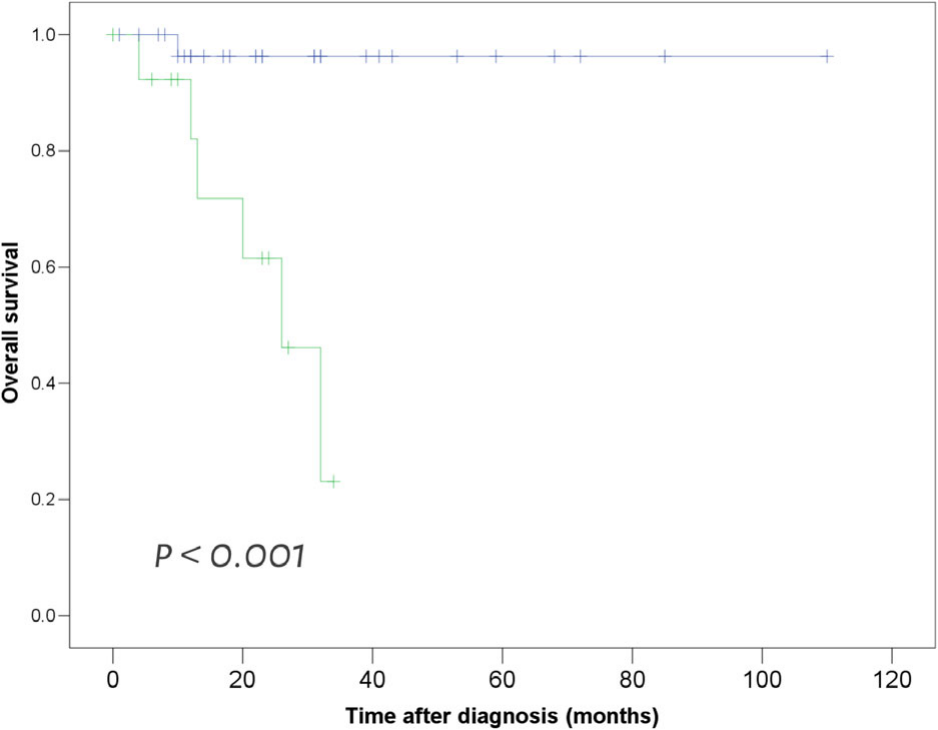
Kaplan‐Meier survival curves show overall survival of patients with invasive mucinous adenocarcinomas of the lung. Curves demonstrate that patients with spontaneous regression of airspace opacities (SRA) show poorer survival compared to those without SRA. 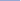
no‐SRA, 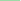
SRA, 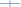
Censored, 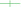
Censored.

Seven patients died during follow‐up, of which six were from the SRA group (*P* = 0.001). All seven patients died due to cancer progression. Mean OS was 86.6 months (range, 0–110 months) for all IMAs, 24.0 months (range, 0–24 months) for the SRA group and 106.3 months (range, 0–110 months) for the no‐SRA group (*P* < 0.001). The poorer OS of IMAs in the SRA group is demonstrated in the survival curves (Fig 4).

### CT findings

A total of 12 (85.7%) out of 14 IMAs in the SRA group were pneumonic type and 30 (93.7%) out of 32 in the no‐SRA group IMAs were nonpneumonic type on initial CT examination (*P* < 0.001). GGO component was present on initial CT in nine (64.3%) of 14 patients in SRA group and 12 (37.5%) of 32 patients in the no‐SRA group. However, because 93.7% of the no‐SRA group were nonpneumonic type, most of their GGO component was seen as part of a subsolid nodule, whereas most of the GGO component consisted of ill‐defined GGO in the vicinity of pneumonic‐type consolidation in the SRA group. The mean size of the IMA in the SRA group was 3.3 ± 1.9 cm (range 0.6–6.7 cm) and that of pneumonic type was 9.5 ± 4.4 cm on initial CT scan (range 6.5–18.1 cm) (*P* < 0.001). IMAs in the SRA group more frequently (*P* = 0.011) showed an ill‐defined margin (10 [71.4%] out of 14 in the SRA group vs. 10 [31.3%] out of 32 in the no‐SRA group) and multifocality (8 [57.1%] out of 14 in the SRA group vs. 1 [6.2%] out of 32 in the no‐SRA group; *P* < 0.001) compared to IMAs in the no‐SRA group. Initial CT findings of IMAs are summarized in Table 2.

**Table 2:** Initial CT findings in 46 patients with invasive mucinous adenocarcinoma of the lung

Characteristics	No‐SRA group (*n* = 32)	SRA group (*n* = 14)	*P*‐value
CT type, n (%)			<0.001*
Pneumonic type	2 (6.3)	12 (85.7)	
Nonpneumonic type	30 (93.7)	2 (14.3)	
GGO component, n (%)			0.093
Absent	20 (62.5)	5 (35.7)	
Present	12 (37.5)	9 (64.3)	
Distribution, n (%)			<0.001*
Solitary	31 (96.9)	6 (42.9)	
Multifocal	1 (6.2)	8 (57.1)	
Mean size ± SD, cm^†^	3.3 ± 1.9	9.5 ± 4.4	<0.001*
Margin, n (%)^‡^			0.011*
Ill‐defined	10 (31.3)	10 (71.4)	
Well‐defined	22 (68.7)	4 (28.6)	
Pleural effusion, n (%)	2 (6.3)	2 (14.3)	0.393

*P < 0.05 indicated by bold italics.

^†^ Size of the largest lesion, if multifocal.

^‡^ Margin of the largest lesion, if multifocal.

GGO, ground‐glass opacity; SD, standard deviation; SRA, spontaneous regression of airspace opacities.

In the SRA group, the median number of follow‐up CTs was 13, and median follow‐up interval between CTs was 44 days. On average (median), approximately three out of 13 follow‐up CTs showed SRAs and the remaining nine follow‐up CTs did not demonstrate SRA. About 66.7% of the CTs that showed SRA were taken after receiving anticancer therapy since the last CT examination. The total number of follow‐up CTs, median follow‐up interval between CTs, and number of CTs that showed, or did not show, SRA with or without receiving anticancer therapy since the last CT for each patient in the SRA group are summarized in Table 3.

**Table 3:** Total number of follow‐up CTs, median follow‐up interval between CTs, and number of CTs that showed or did not show spontaneous regression of airspace opacities (SRA) with or without receiving anticancer therapy since last CT for each patient in the SRA group

	Number of CTs that showed SRA			Number of CTs that did not show SRA				
Patient	Number of CTs taken after receiving anticancer therapy since last CT	Number of CTs taken without receiving anticancer therapy since last CT	Total number of CTs	Number of CTs taken after receiving anticancer therapy since last CT	Number of CTs taken without receiving anticancer therapy since last CT	Total number of CTs	Total number of follow‐up CTs	Follow‐up interval between CTs^†^, median (range)
1	3	0	3	2	2	4	7	52 (18–131)
2	2	3	5	2	5	7	12	33 (6–68)
3	1	6	7	1	6	7	14	57 (15–239)
4	3	3	6	6	7	13	22	29 (7–61)
5	1	3	4	9	4	13	17	63 (17–181)
6	3	0	3	14	2	16	19	45 (13–91)
8	0	2	2	0	1	1	3	41 (35–92)
9	4	0	1	11	1	12	16	42 (11–63)
11	2	0	2	6	6	12	12	38 (7–63)
12	0	1	1	0	1	1	2	164 (21–306)
13	3	1	4	1	1	2	6	30 (19–60)
14	1	1	2	21	0	21	23	42 (12–66)
15	2	0	2	10	1	11	13	47 (28–84)
16	4	0	4	1	3	4	8	48 (20–63)
Median	2	1	3	4	2	9	13	44 (29–164)

^†^ Time in days.

SRA, spontaneous regression of airspace opacities.

### Pathological analysis

Pathological diagnosis was made using needle biopsy specimen in 11 patients (23.9%), surgical specimens in 18 patients (39.1%), and both needle biopsy and surgical specimens in 17 (37.0%) patients. Two (6.3%) of 32 no‐SRA group patients were diagnosed only using needle biopsy specimens, whereas nine (64.3%) of 14 SRA group patients were diagnosed using only biopsy specimens (*P* < 0.001). Among the cases available for molecular studies, *EGFR* mutation was found in one out of 40 cases (2.5%, exon 19 deletion) and *KRAS* mutation was found in seven out of 16 cases (43.8%). G12V was the most common type of *KRAS* mutation (five out of seven cases), and the remaining were G12C and G13D mutations.

**Figure 5:**
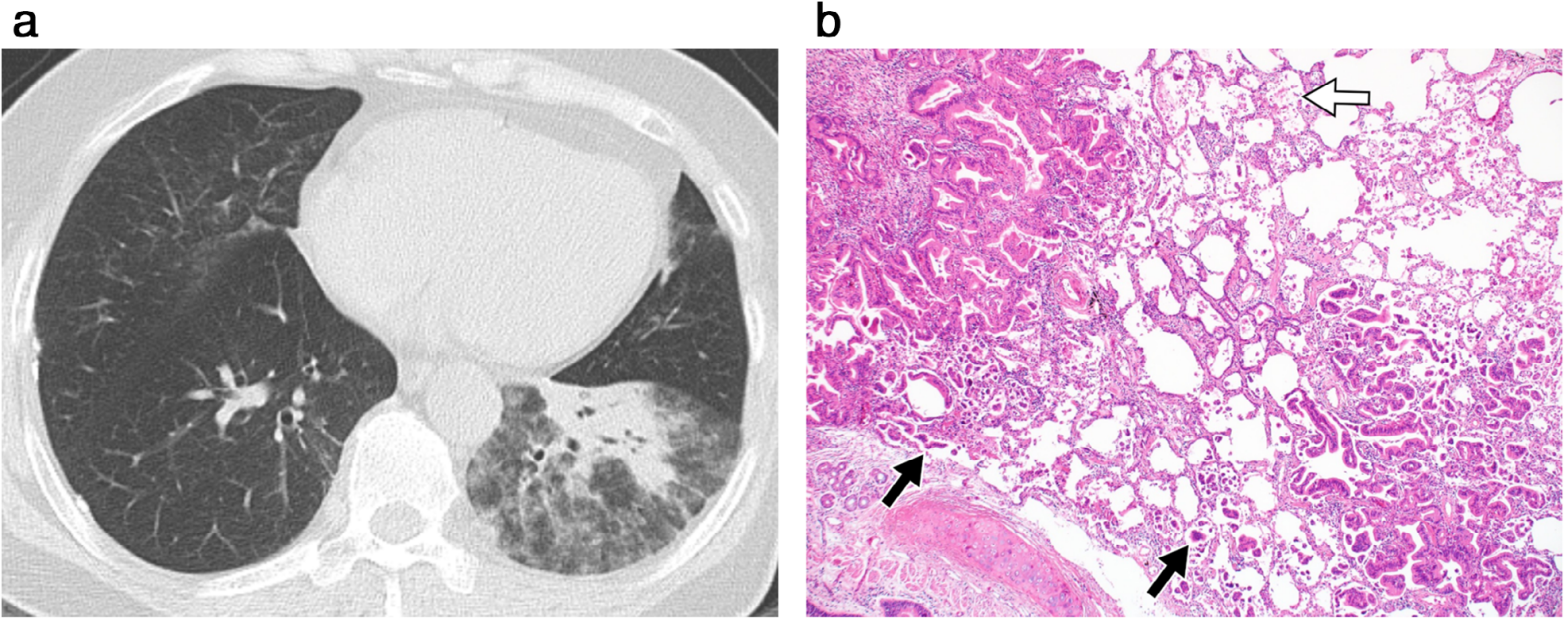
(**a**) Axial chest CT image (section thickness, 3.0 mm) of a 76‐year‐old female patient with pneumonic‐type invasive mucinous adenocarcinoma show irregular consolidation with surrounding area of ground‐glass opacity in the left lower lobe. (**b**) Photomicrograph of the lobectomy specimen corresponding to the area of ground‐glass opacity shows numerous tumor nodules (left upper and right lower areas). Between the nodules, intra‐alveolar macrophages (white arrow) and tiny tumor cell nests (black arrows) floating into the intra‐alveolar mucin are observed. Organizing pneumonia or fibrosis is not observed in this area (hematoxylin and eosin stained, x40).

The area of GGO in IMAs of these patients on CT images correlated histopathologically with several components: IMA growing on the alveolar wall, organizing pneumonia, mucinous material with macrophages and tiny tumor cell nests in the alveolar space, and fibrosis (Figs 3 and 5). The proportion of each component varied area‐by‐area and patient‐by‐patient; eg, some areas matched with more mucinous material while other areas showed more components of organizing pneumonia. In other words, although seen similarly as GGO on CT, the proportion of components seen on pathological examination differed on a patient‐by‐patient basis. The area of consolidation on CT images corresponded to the areas of densely packed tumor glands with focal fibrosis (Fig 3).

## Discussion

Our study demonstrated that about 30% of IMAs of the lung showed what we describe as SRA on serial CT findings, which is mostly observed in pneumonic‐type IMAs. There has been one case report which has described the same phenomenon, although the authors used the term “fluctuating extent” instead of SRA.^4^ However, to the best of our knowledge, our study is the first to systematically investigate this phenomenon in IMAs. By correlating the CT images with histopathological analysis of pneumonic‐type IMAs in the SRA group, we can substantiate this hypotheses. Pneumonic‐type IMAs, seen as consolidation and GGOs with ill‐defined border on CT images, are composed of tumor and nontumor components. GGO areas are composed of tumor growing on the alveolar wall, organizing pneumonia, mucinous material with macrophages and tumor nests floating in the alveolar space, and fibrosis. Our observation is similar to that made in another study, which stated that IMAs “produce abundant extracellular mucin that typically fills the alveoli and often contains viable detached neoplastic cells within the intraalveolar mucus”.^12^ The areas consisting of organizing pneumonia and mucinous material with macrophages floating in the alveolar spaces are likely to change over time, regardless of anticancer treatment; and this would then appear as SRA on follow‐up CT images.

Our findings on SRA also suggest that a more careful application of RECIST 1.1 is needed in the assessment of tumor response of IMAs. Whether the decrease in size of airspace opacities is a true reflection of tumor response to therapy or a mere decrease in mucinous material or organizing pneumonia cannot be determined radiologically, because SRA is seen regardless of use of anticancer therapy. Therefore, interpreting tumor response in IMAs should be done with caution if the lesion has shown SRA, or is of, pneumonic‐type. What may look like progressive disease or partial response may actually be SRA.

IMAs in the SRA group showed worse prognosis, with shorter OS and higher mortality. Because the SRA group mostly consisted of pneumonic‐type IMAs, our findings are in line with other studies that evaluated resected IMAs and concluded that pneumonic types showed worse prognosis than the nonpneumonic types.^7^, ^8^, ^9^ This may be in part due to the fact that IMAs in the SRA group tended to be significantly more multifocal on initial CT images, automatically classifying them as T4 M1a (stage IV) tumors in many patients. Multifocal airspace opacities may or may not have been entirely due to tumor component and these airspace opacities may come and go. Nevertheless, the shorter OS and higher mortality of these patients corroborate that they are poor prognostic factors. Thus, they were correctly classified as stage IV. In stage III patients, the rate of local recurrence after resection was much higher (100% vs. 11.5%) in the SRA group, although the numbers were too small to give a statistically significant comparison. Considering that airspace opacities, especially GGOs, inconsistently appear and disappear in IMAs that show SRAs, it is possible that extracellular mucin containing small tumor nests may have existed in other lobes without being detected on the preoperative CT. Thus, the undetected small tumor nests with extracellular mucin might appear as GGOs on the later follow‐up CTs. Even if the preoperative CT shows cancer lesion confined to one lobe, the possibility of undetected mucin in other lobes and risk of recurrence should be kept in mind when choosing treatment plans between systemic treatment and surgery for IMAs showing SRAs.

There are several limitations to this study. First, it was a retrospective study with a relatively small sample size. Due to the small sample size, the effect of SRA on OS cannot be concluded with certainty. Second, the treatment methods as well as the types of drugs used for IMA patients who received palliative treatments varied. Also, many patients who received palliative treatments received more than one method of palliative treatment. Difference in palliative treatments could have influenced the treatment outcomes, but tracking all the different combinations of palliative treatments seemed beyond the scope of this study, so we did not investigate the effect of different treatment methods on the outcome. Third, we were able to histopathologically evaluate the nature of GGO and consolidation that comprise SRAs only in about 35% of patients in the SRA group, and they were all pneumonic‐type IMAs; the histopathological nature of SRAs in other patients without surgical specimens, especially those of nonpneumonic‐type IMAs, may differ. Further studies with larger number of patients with surgical specimens for review may be helpful.

In conclusion, IMAs of the lung with SRAs on serial CT review are larger and multifocal, have higher stage, higher mortality, and may show poorer OS. The areas of GGO in SRA‐group IMA consist of tumor growing on the alveolar wall, organizing pneumonia, mucinous material with macrophages and tumor nests floating in the alveolar space, and fibrosis.

## Disclosure

The authors have no conflicts of interest to declare.

## Supporting information


**Appendix S1.** Supporting information
