# Supplementary material for: Invasive mucinous adenocarcinoma of the lung: Serial CT findings, clinical features, and treatment and survival outcomes
Source: Thorac Cancer. 2020 Oct 5;11(12):3463–72. doi: 10.1111/1759-7714.13674 (PMC7705898; doi:10.1111/1759-7714.13674)
Supplement: Supplementary file 1 — Appendix S1. Supporting information [file TCA-11-3463-s001.docx]

**Supplementary Figures**


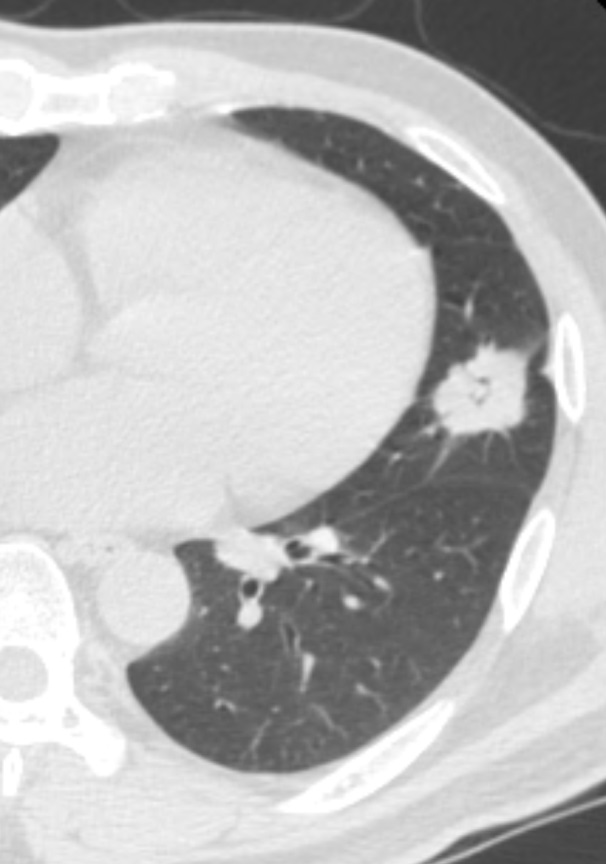
**
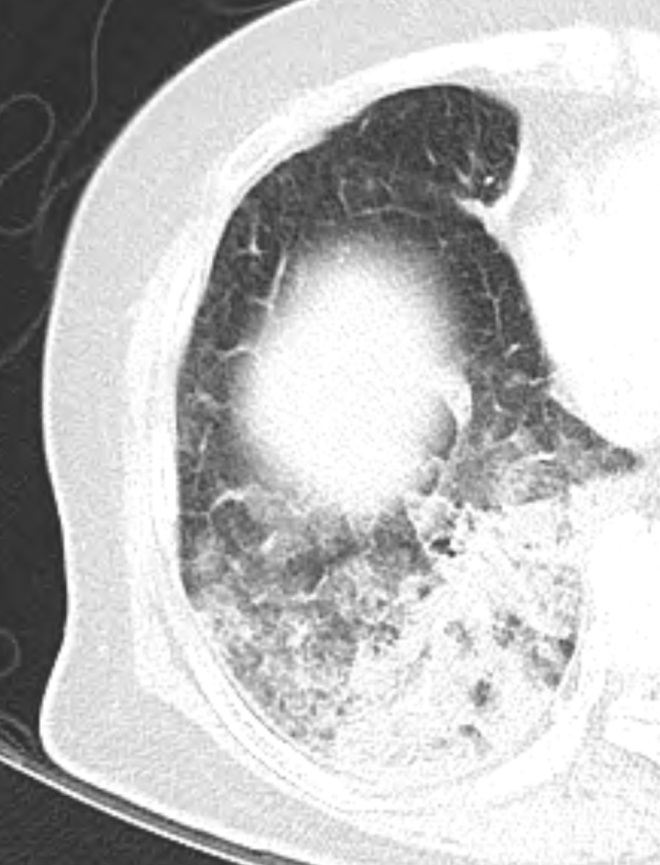
**

**a. b.**

**Figure S1 –** Typical examples of pneumonic and non-pneumonic type invasive mucinous adenocarcinoma **(a)** Axial chest CT image (section thickness, 3.0 mm) of a 50-year-old male patient shows a typical non-pneumonic type invasive mucinous adenocarcinoma seen as an oval, lobulated nodule with spiculations in the lingular divion of left upper lobe. **(b)** Axial chest CT image (section thickness, 3.0 mm) of a 74-year-old female patient shows a typical example of pneumonic type invasive mucinous adenocarcinoma, seen as ill-defined consolidation and ground-glass opacity in the dependent portion of right lower lobe.
